# Liraglutide enhances the effect of checkpoint blockade in lung and liver cancers through the inhibition of neutrophil extracellular traps

**DOI:** 10.1002/2211-5463.13499

**Published:** 2024-06-28

**Authors:** Duo Chen, Hongxin Liang, Luyu Huang, Haiyu Zhou, Zheng Wang

**Affiliations:** ^1^ Department of Respiratory and Critical Care Medicine, Beijing Institute of Respiratory Medicine, Beijing Chao‐Yang Hospital Capital Medical University Beijing China; ^2^ Department of Thoracic Surgery, Guangdong Provincial People's Hospital Guangdong Academy of Medical Sciences Guangzhou China; ^3^ Department of Surgery, Competence Center of Thoracic Surgery Charité Universitätsmedizin Berlin Germany

**Keywords:** liraglutide, liver cancer, lung cancer, neutrophil extracellular traps, PD‐1

## Abstract

Glucagon‐like peptide‐1 (GLP‐1) regulates glycemic excursions by augmenting insulin production and inhibiting glucagon secretion. Liraglutide, a long‐acting GLP‐1 analog, can improve glycemic control for treating type 2 diabetes and prevent neutrophil extravasation in inflammation. Here, we explored the role of liraglutide in the development and therapy of murine lung and liver cancers. In this study, liraglutide substantially decreased circulating neutrophil extracellular trap (NET) markers myeloperoxidase, elastase, and dsDNA in Lewis lung cancer (LLC) and Hepa1‐6 tumor‐bearing mice. Furthermore, liraglutide downregulated NETs and reactive oxygen species (ROS) of neutrophils in the tumor microenvironment. Functionally, *in vitro* experiments showed that liraglutide reduced NET formation by inhibiting ROS. In addition, we showed that liraglutide enhanced the anti‐tumoral efficiency of programmed cell death‐1 (PD‐1) inhibition in LLC and Hepa1‐6 tumor‐bearing C57BL/6 mice. However, the removal of NETs significantly weakened the antitumor efficiency of liraglutide. We further demonstrated that the long‐term antitumor CD8^+^ T cell responses induced by the combination therapy rejected rechallenges by respective tumor cell lines. Taken together, our findings suggest that liraglutide may promote the anti‐tumoral efficiency of PD‐1 inhibition by reducing NETs in lung and liver cancers.

## Graphical abstract

Liraglutide substantially decreases circulating and tumor‐infiltrated neutrophil extracellular traps by inhibiting reactive oxygen species production. In addition, liraglutide enhances the anti‐tumoral efficiency of programmed cell death‐1 inhibition in tumor‐bearing mice. Furthermore, combination therapy improves IFN‐γ release by CD8^+^ T cells. Finally, the long‐term antitumor CD8^+^ T cell response induced by the combination therapy rejects tumor rechallenges.

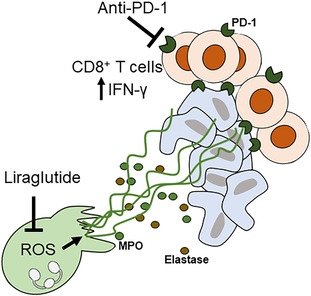

**Abbreviations**
GLP‐1: glucagon‐like peptide‐1
ICIs: immune checkpoint inhibitors
LLC: Lewis lung cancer
NETs: neutrophil extracellular traps
PD‐1: programmed cell death‐1
PD‐L1: programmed cell death‐ligand 1
ROS: reactive oxygen species
STR: surgical tumor resection

Glucagon‐like peptide‐1 (GLP‐1) could regulate glycemic excursions by augmenting insulin production and inhibiting glucagon secretion [1]. Liraglutide, a long‐acting GLP‐1 analog with 97% structural homology to the native hormone [2], binds to the GLP‐1 receptor, displaying a similarly broad range of activities relevant to improving glycemic control for treating type 2 diabetes. Additionally, GLP‐1 receptor agonists, including liraglutide, are safe and not associated with an increased risk of pancreatic and breast cancer [3, 4, 5].

Furthermore, liraglutide presented anticancer effects on breast cancer cells [3]. However, the influence of liraglutide on other cancers is yet to be reported. In addition, GLP‐1 also has immune modulatory roles. It was reported that GLP‐1 stimulation regulated eosinophil activation in allergic asthmatic subjects [6] and decreased neutrophil activation in rodents [7]. Liraglutide also demonstrated promising anti‐inflammatory and immunomodulatory activities [8]. It has been identified that liraglutide alleviates lipopolysaccharide‐induced acute lung injury by preventing neutrophil extravasation [2, 9].

Neutrophils have been shown to mediate direct and indirect protumor and antitumor effects during early tumor initiation and growth [10]. Although an early antitumorigenic role of neutrophils was reported by some researchers [11, 12, 13, 14, 15, 16], neutrophils have been shown to mediate mostly pro‐tumoral effects in the tumor microenvironment [17]. Neutrophils could desorb neutrophil extracellular traps (NETs) in a process sometimes termed NETosis, although cell death is not necessarily required for NETs formation [18, 19, 20]. NETs are web‐like chromatin structures decorated with proteins responsible for trapping and killing extracellular pathogens, representing an antimicrobial mechanism of neutrophilic granulocytes. In addition, neutrophils are vital in promoting tumor growth and progression. Increasing evidence herein points to NETs as potential mediators [21, 22]. NETs could also shield tumor cells, physically obstructing contact with CD8^+^ T cells and natural killer cells in Lewis lung carcinoma, thus fostering tumor spread at distant sites [21, 22].

The laboratory and clinical evidence supported that NETs degradation, such as neutrophil elastase inhibitor and DNase 1, could mitigate NET‐dependent cancer progression and treatment resistance [23, 24, 25]. Eventually, NETs inhibition could enhance the response of programmed cell death‐1 (PD‐1) inhibitors in pancreatic cancer [26]. The recent development of immune checkpoint inhibitors (ICIs) targeting PD‐1 and programmed cell death‐ligand 1 (PD‐L1) have contributed to improvements in the prognosis of many cancer patients [27, 28, 29]. However, the unprecedented response rates and survival of ICIs only occur in 15–40% of patients [30, 31]. Therefore, the relationship between responses to ICIs and key host factors, such as immunologic statuses, needs to be thoroughly investigated. However, the modulation of liraglutide in neutrophils and immunotherapy in the context of cancers is unknown.

In this study, we found that in lung and liver tumor mice, liraglutide reduced the NETs formation by inhibiting reactive oxygen species (ROS) production. Furthermore, the reduction of NETs by liraglutide enhanced the anti‐tumoral response of PD‐1 inhibition, and the combination therapy induced long‐term antitumor CD8^+^ T cell responses. Hereby, this study revealed that liraglutide improved the anti‐tumoral efficiency of PD‐1 inhibition against lung and liver cancers, providing therapy candidates for tumor treatment.

## Materials and methods

### Animal studies

Five‐week‐old male C57BL/6 mice were purchased from Capital Medical University Animal Laboratories (Beijing, China). Subcutaneous inoculation of Lewis lung cancer (LLC) or Hepa1‐6 cells was made at 5 × 10^6^ cells in 100 μL, and the mice were divided into several groups. One week later, they accepted intraperitoneal injection of PBS, liraglutide (400 μg·kg^−1^·day^−1^; Novo Nordisk) [32], anti‐mouse PD‐1 (250 μg per mice, twice/week; Clone: RMP1‐14; BioCell) [33] or the combination of liraglutide and anti‐PD‐1 for 2 weeks. To remove NETs in C57BL/6 mice, DNase I (5 mg·kg^−1^) [34] was administered intraperitoneally starting 1 week after tumor cell inoculation, and DNase I was injected daily for 2 weeks.

For surgical tumor resection (STR) and tumor rechallenge, STR was operated in PBS or Liraglutide+anti‐mouse PD‐1 (Liraglutide+αPD‐1) treated tumor mice under isoflurane anesthesia at tumor volume > 200 mm^3^ and a minimum of 15 days after tumor cell inoculation [35]. Tumors were exposed for excision and then disconnected with a disposable cautery to prevent bleeding, and surgical incisions were closed with wound clips. Carprofen (5 mg·kg^−1^, s.c.) was used for analgesia prior to and after STR.

Tumor sizes were measured every 2 days and were calculated using the formula: volume = 0.5 × length × width^2^.

All animal studies were approved by the Animal Welfare and Research Ethics Committee of Capital Medical University (No. 2018‐2‐8‐47). Furthermore, all animal experiments were performed in accordance with the institutional and national regulations.

### Cell lines

Murine Lewis lung cancer cell LLC and liver cancer cell Hepa1‐6 were cultured in Dulbecco's Modified Eagle's Medium containing 10% FBS and 1% streptomycin–penicillin (10 000 U·mL^−1^) (all: Corning, New York, NY, USA) in a humidified incubator at 37 °C containing 5% CO_2_. LLC and Hepa1‐6 cells were from the Shanghai Cell Biology Institute of the Chinese Academy of Sciences (Shanghai, China).

### Circulating molecules and NETs biomarkers

Commercially available ELISA kits were used to measure murine plasma GM‐CSF (GM‐CSF Mouse ELISA Kit; Thermo Fisher Scientific, Waltham, MA, USA), VEGF‐A (VEGF‐A Mouse ELISA Kit; Thermo Fisher Scientific), MMP‐9 (Mouse MMP9 ELISA Kit; Abcam), myeloperoxidase (Myeloperoxidase Mouse ELISA Kit; Thermo Fisher Scientific), and elastase (Mouse Neutrophil Elastase ELISA Kit; Abcam) according to the manufacturer's instructions. The serum cell‐free double strand (ds) DNA concentration was measured using the phenol/chloroform preparation method. The separated DNA pellet was dissolved in UltraPure DNase/RNase‐Free Distilled Water (Thermo Fisher Scientific). The dsDNA measurement was performed using the ultramicrospectrophotometer NANODROP ONE (Thermo Fisher Scientific), 1 μL of the sample was used for measurement, and DNA concentration was determined by measuring the absorbance at 260 nm.

### Enrichment of neutrophils

To enrich tumor infiltrated neutrophils, the tumor tissues were minced with surgical scissors and dissociated in RPMI 1640 medium containing collagenase type I (0.05 mg·mL^−1^; Sigma‐Aldrich, Burlington, MA, USA) and DNase I (0.01 mg·mL^−1^; Roche, Switzerland), then incubated at 37 °C for 10 min, followed by being filtered through cell strainers that are available in 70 μm sizes for the acquisition of single cells. Subsequently, neutrophils were enriched using the EasySep™ Mouse Neutrophil Enrichment Kit (STEMCELL Technologies).

### NETs assay

The enriched murine neutrophils were seeded in a 24‐well tissue culture plate at 2 × 10^5^ neutrophils/well. The NET formation assays refer to the previous description [26]. Briefly, the neutrophils were allowed to adhere for ∼ 20 min and were then incubated with liraglutide (100 mm) for 1 h [36], followed by the stimulation with PMA (Sigma‐Aldrich) at 25 nm for 2 h. Thereafter, 200 nm SYTOX Green (SYTOX™ Green Nucleic Acid Stain; Thermo Fisher Scientific) was carefully added to the plate for staining for 15 min. Then, the cells were imaged with a Zeiss Vert.A1 fluorescence microscope. For NETs quantification, the fluorescence intensity was measured using Synergy2 Multi‐Mode Microplate Reader (BioTek). Cells lysed with 0.5% Triton X‐100 were considered 100% DNA release.

### Detection of intracellular ROS production

The intracellular ROS levels were measured using the Total Reactive Oxygen Species Assay Kit (Thermo Fisher Scientific), and the fluorescence was monitored with flow cytometry assay.

### Detection of IFN‐γ secretion by tumor‐infiltrating CD8 ^+^ T cells by ELISA

CD8^+^ T cells from tumor‐draining lymph nodes, spleens, or tumor tissues were enriched from LLC or Hepa1‐6 tumor‐bearing C57BL/6 mice using EasySep™ Mouse CD8^+^ T Cell Isolation Kit (STEMCELL Technologies). According to the previous description [37], the enriched CD8^+^ T cells (1 × 10^5^ cells) were mixed with irradiated LLC or Hepa1‐6 tumor cells (1 × 10^3^ cells) and incubated in 96‐well culture plates for 48 h in a cell culture incubator. The supernatant was collected and assayed for IFN‐γ secretion by ELISA according to the manufacturer's instructions (IFN gamma Mouse ELISA Kit; Thermo Fisher Scientific).

### Statistical analysis

Data were expressed as the mean ± SD. Statistical comparison was analyzed using the two‐tailed Student's *t*‐test or two‐way ANOVA. *P* < 0.05 (*) was considered statistically significant.

## Results

### Liraglutide decreased circulating NETs markers in tumor mice

**Fig. 1:**
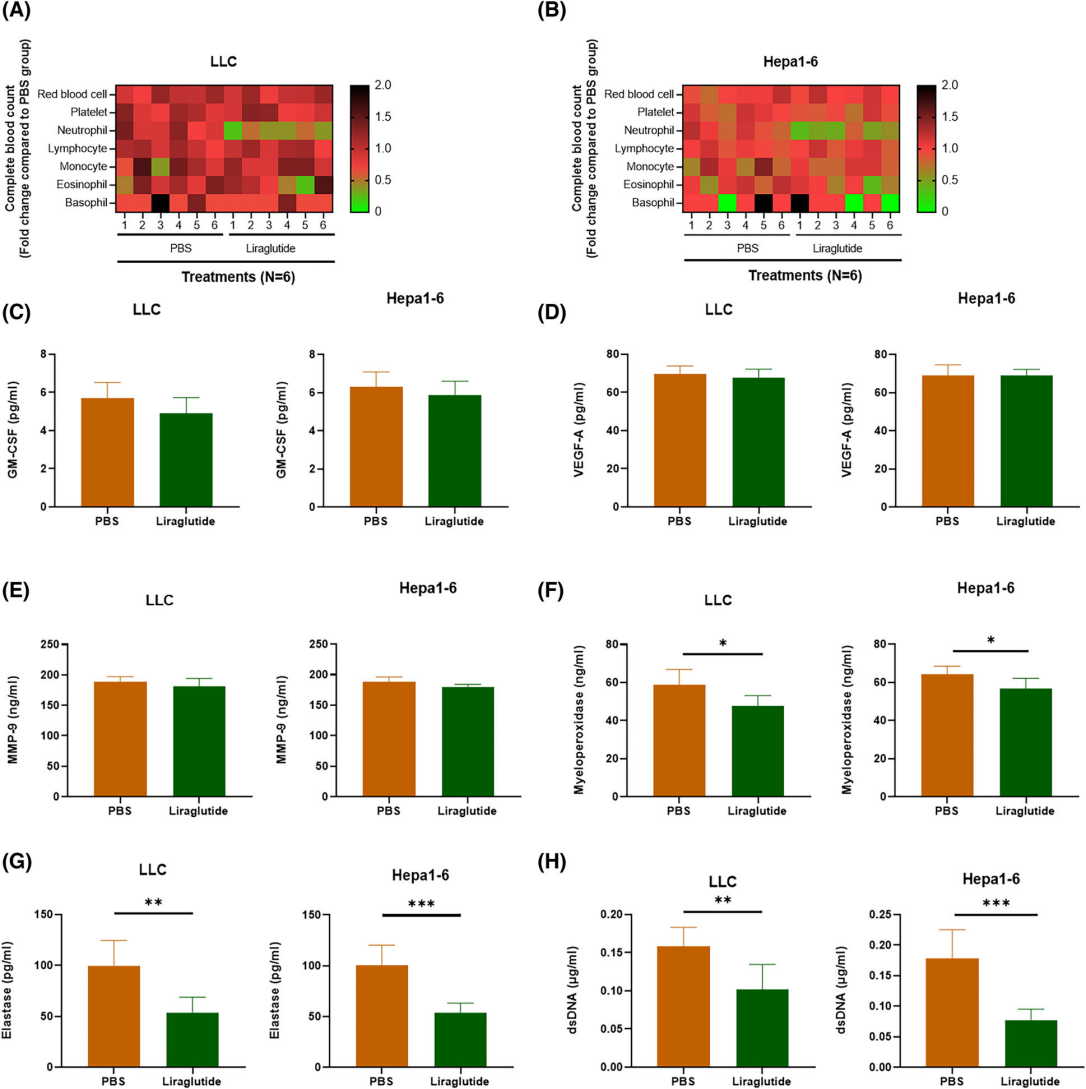
Liraglutide inhibits tumor progression through neutrophils. Measure blood cells in (A) LLC and (B) Hepa1‐6 cells bearing C57BL/6 mice using complete blood count. Concentrations of circulating (C) GM‐CSF, (D) VEGF‐A, (E) MMP‐9, (F) myeloperoxidase, and (G) elastase in mice were measured using ELISA. (H) Circulating dsDNA was purified and measured using an ultramicrospectrophotometer. Statistical significance was determined with *t*‐test. *N* = 6 mice for every group. Nonparametric tests were utilized to assess statistical significance between different treatment groups. *P < 0.05, **P < 0.01, ***P < 0.001. Error bars represent SD.

To explore the influence of liraglutide on tumor progression, we constructed the LLC and liver cancer models. The lung or liver cancer models were established by subcutaneous injection of LLC or Hepa1‐6 cells into male C57BL/6 mice. One week after the tumor implant, the mice were intraperitoneally injected with PBS or liraglutide every day for 2 weeks. To explore the impact of liraglutide on blood cells in lung and liver cancers, we first conducted complete blood count. The results showed that liraglutide significantly decreased neutrophils in both tumor models (Fig. 1A,B). To assess the impact of liraglutide on neutrophils' function, we collected plasma from the LLC and Hepa1‐6 bearing C57BL/6 mice after therapy with either liraglutide or PBS. Then we measured the concentration of circulating GM‐CSF, VEGF‐A, MMP‐9, myeloperoxidase, and elastase using ELISA and detected dsDNA using the spectrometer. The results showed that liraglutide presented a mild impact on GM‐CSF (Fig. 1C), VEGF‐A (Fig. 1D), and MMP‐9 (Fig. 1E) compared to PBS‐treated mice. Meanwhile, mice in the liraglutide group experienced a significant reduction in the concentrations of myeloperoxidase (LLC: −19%; Hepa1‐6: −12%) (Fig. 1F), elastase (LLC: −48%; Hepa1‐6: −48%) (Fig. 1G), and dsDNA (LLC: −45%; Hepa1‐6: −57%) (Fig. 1H). Interestingly, circulating myeloperoxidase, elastase, and dsDNA are NETs biomarkers, indicating that liraglutide might reduce NETs in tumor‐bearing mice.

### Liraglutide reduced NETs and ROS of tumor‐infiltrated neutrophils

**Fig. 2:**
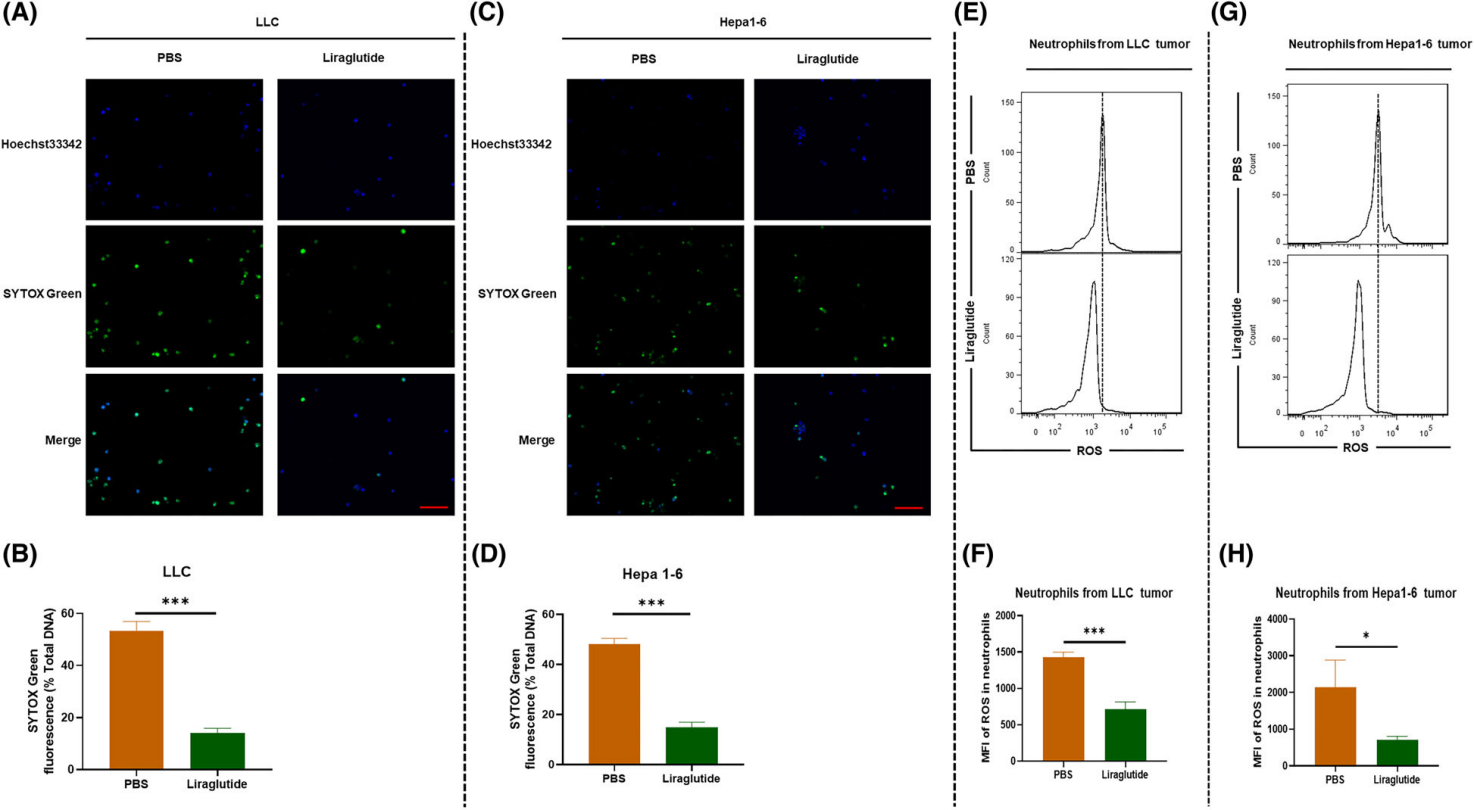
Liraglutide reduced tumor‐infiltrated NETs. The LLC and Hepa1‐6 cells bearing C57BL/6 mice were treated using PBS or liraglutide, and neutrophils were enriched for *in vitro* study. Representative images of tumor‐derived NETs from (A) LLC and (C) Hepa1‐6 cells bearing C57BL/6 mice. Blue is nuclear stained by Hoechst 33342. Green is DNA stained by SYTOX Green. Quantification of tumor‐derived NETs from (B) LLC and (D) MC38 cells bearing C57BL/6 mice were conducted using a fluorescence microplate reader (*n* = 3). The tumor infiltrated neutrophils from (E) LLC and (G) Hepa1‐6 cells bearing C57BL/6 mice were stained with total reactive oxygen species (ROS) assay kit, ROS production was analyzed by flow cytometry, and the green fluorescence of ROS was detected using FITC channel. Mean fluorescence intensity (MFI) of ROS in tumor‐derived neutrophils from (F) LLC and (H) MC38 cells bearing C57BL/6 mice were conducted using a fluorescence microplate reader (*n* = 3). Nonparametric tests were utilized to assess statistical significance between different treatment groups. *P < 0.05, ***P < 0.001. Error bars represent SD. Scale bar: 400 μm (A, C).

To further explore the influence of liraglutide on NETs, we enriched tumor‐infiltrated neutrophils from the tumor models after liraglutide treatment, and then detected NETs using SYTOX Green staining. The results showed that, as compared to PBS groups, liraglutide treatment significantly reduced NETs in both LLC (Fig. 2A,B) and Hepa1‐6 (Fig. 2C,D) tumor models. Published studies have suggested that ROS is essential for NETs formation [21, 38], and liraglutide could downregulate the generation of ROS [39, 40, 41]. Therefore, we further explored the role of ROS in inhibiting NET by liraglutide. We detected ROS in neutrophils from the tumor tissues using flow cytometry, and the results showed that, as compared to PBS groups, liraglutide treatment significantly reduced ROS production in both LLC (Fig. 2E,F) and Hepa1‐6 (Fig. 2G,H) tumor models. The results indicate that liraglutide reduced NETs and ROS of tumor‐infiltrated neutrophils.

### Liraglutide reduced NETs formation through the inhibition of ROS

**Fig. 3:**
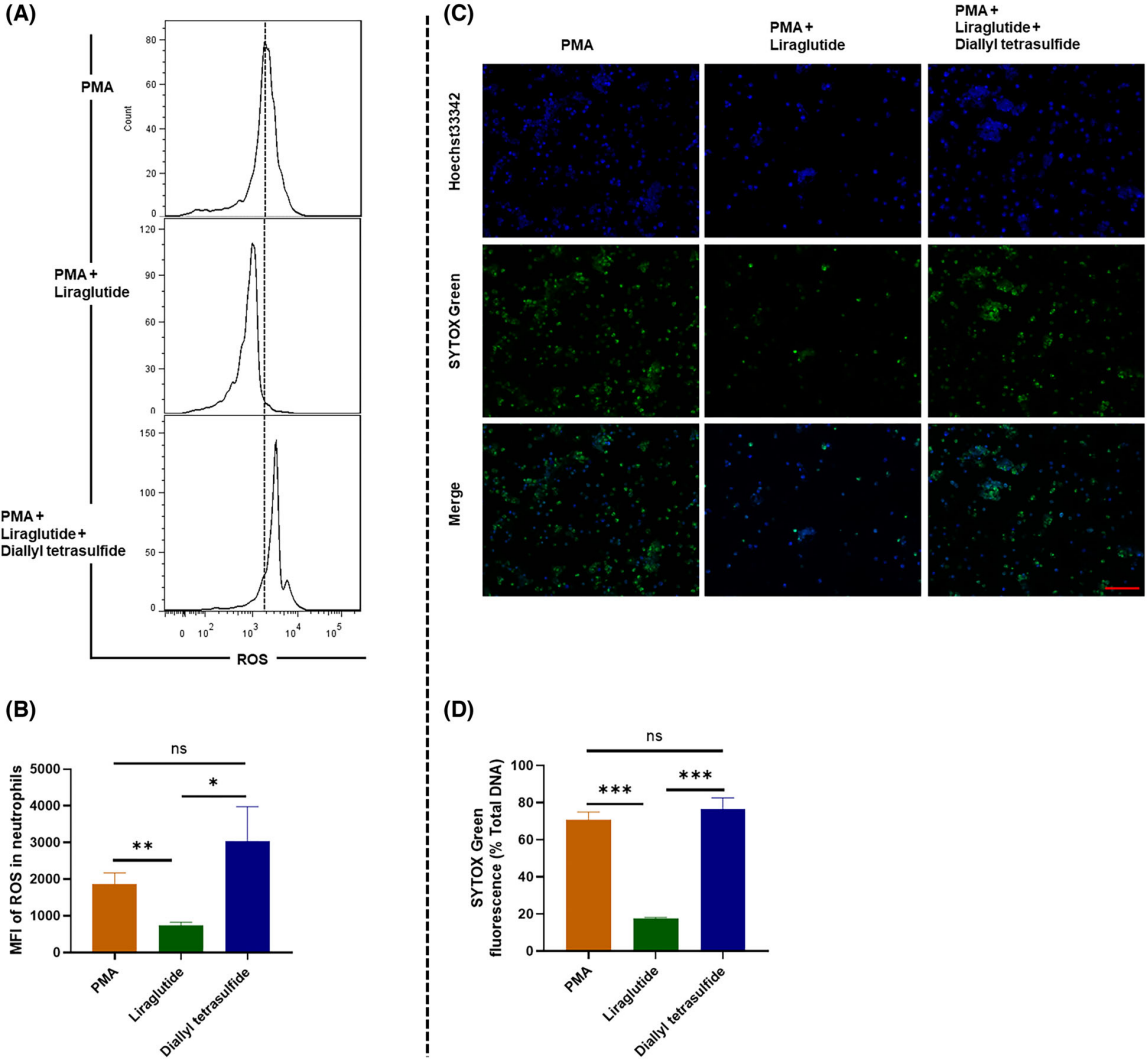
Liraglutide reduced NETs formation by inhibiting ROS production. The enriched naïve murine neutrophils were incubated with the liraglutide (100 mm) for 1 h, followed by the stimulation with PMA (Sigma‐Aldrich) at 25 nm for 2 h, or incubated with ROS inducer diallyl tetrasulfide. (A) Representative flow cytometry assay of ROS levels and (B) statistical analysis of mean fluorescence intensity (MFI) (*n* = 3). (C) NETs were detected by Hoechst 33342 and SYTOS Green dye. Scale bar: 400 μm. (D) Quantification of tumor‐derived NETs using a fluorescence microplate reader (*n* = 6). Nonparametric tests were utilized to assess statistical significance between different treatment groups. *P < 0.05, **P < 0.01, ***P < 0.001. ns, non‐significant. Error bars represent SD. Triple assays were performed.

We detected ROS in liraglutide‐treated neutrophils by flow cytometry, finding that ROS production was significantly decreased after liraglutide treatment (Fig. 3A,B). According to the previous description [42], we induced NETs by stimulating enriched murine neutrophils with PMA. As a result, we found that pre‐incubation with liraglutide significantly decreased PMA‐induced NETs (Fig. 3C,D). However, applying the ROS inducer Diallyl tetrasulfide in the supernatant of neutrophils treated by PMA and liraglutide led to a significant rescue of NETs formation liraglutide reduced. These results demonstrate that ROS mediated the induction of NETs formation by liraglutide.

### Combining liraglutide and PD‐1 blockade demonstrates enhanced antitumor efficacy

**Fig. 4:**
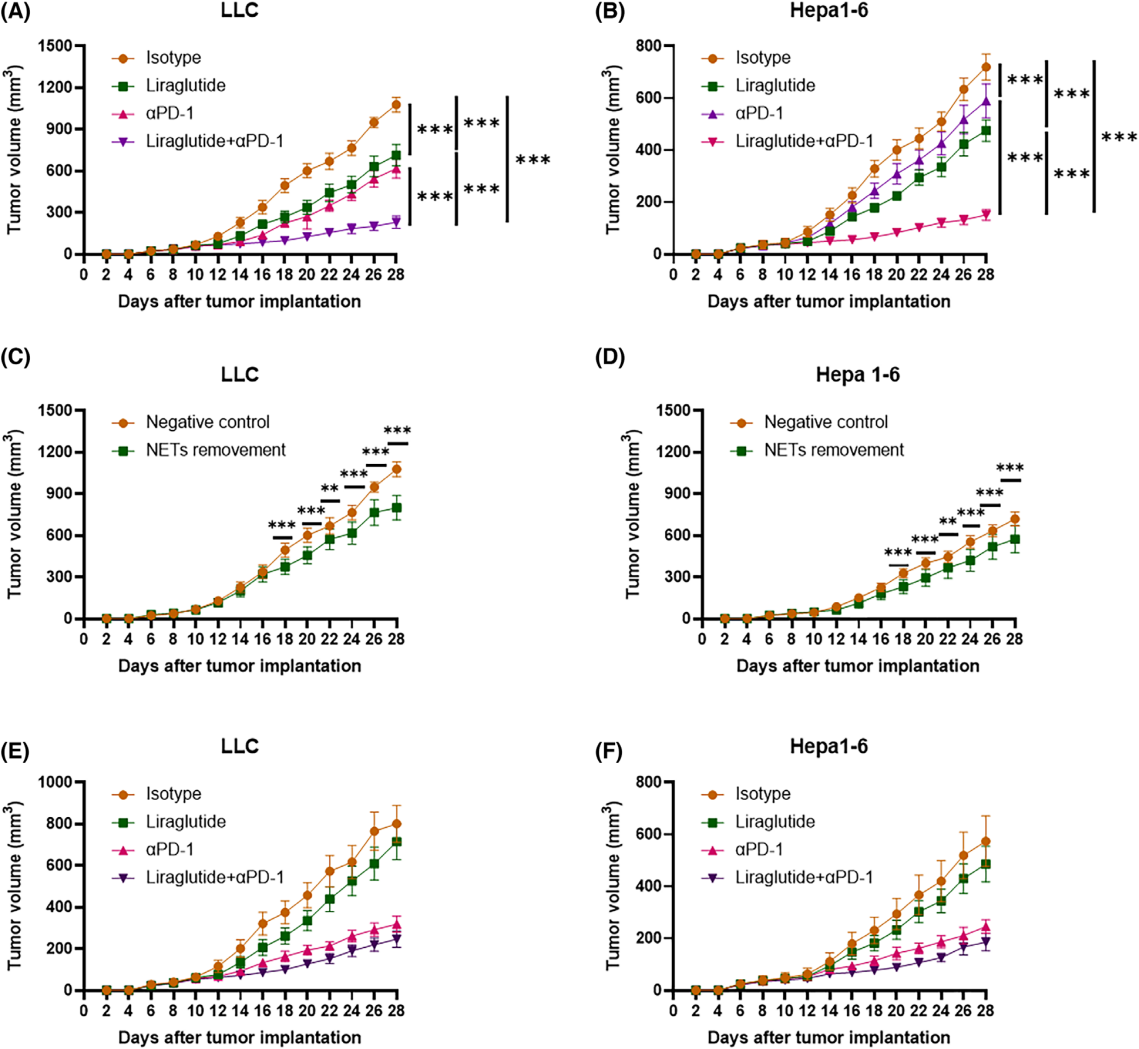
Liraglutide enhanced the antitumor efficacy of PD‐1 blockade. Tumor volumes were monitored in (A) LLC or (B) Hepa1‐6 allograft mice treated by IgG isotype control (isotype), anti‐PD‐1 mAb (αPD‐1), liraglutide, or liraglutide and anti‐PD‐1 mAb (liraglutide+αPD‐1). (C) LLC and (D) Hepa1‐6 tumor volumes for the NETs‐removed C57BL/6 mice. NETs were removed using DNase I (5 mg·kg^−1^). (E) LLC and (F) Hepa1‐6 tumor volumes for the NETs‐removed C57BL/6 mice accepting isotype, αPD‐1, liraglutide, or liraglutide+αPD‐1 treatments. NETs were removed using DNase I (5 mg·kg^−1^). Tumor volume = 0.5 × length × width^2^. Statistical significance was determined with two‐way ANOVA tests. *N* = 12 mice for every group. Nonparametric tests were utilized to assess statistical significance between different treatment groups. **P < 0.01, ***P < 0.001. Error bars represent SD.

Because it was documented that NETs inhibition improves the antitumoral effect of PD‐1 blockade in pancreatic cancer [26], we explored whether this phenomenon exists in lung and liver cancers. Based on the previous results, we further investigated whether liraglutide influenced the efficacy of anti‐PD‐1 (αPD‐1) in treating cancers. We randomized LLC or Hepa1‐6 allograft mice into four groups that received the following treatments: (a) IgG isotype control, (b) αPD‐1, (c) Liraglutide, (d) Liraglutide+αPD‐1. Although liraglutide or αPD‐1 could weaken tumor development, the combination of liraglutide and anti‐PD‐1 showed significantly higher efficiency in restricting tumor growth (Fig. 4A,B). To further validate the roles of NETs in tumor inhibition by liraglutide, we removed NETs in LLC and Hepa1‐6 bearing mice using DNase I. The measurement of circulating dsDNA from the mice confirmed specific deletion of NETs (Fig. S1). NETs are involved in the enhancement of inflammation in autoimmune diseases [43], like systemic lupus erythematosus [44], rheumatoid arthritis [45], psoriasis [46], and also inflammation, such as pulmonary diseases [47]. Multiple research projects indicate the essential roles of IL‐6, IL‐10, IL‐21, and IL‐17 in the pathogenesis of autoimmune diseases [48, 49, 50]. Based on these reports, we measured the concentrations of peripheral IL‐6, IL‐10, IL‐21, and IL‐17 using ELISA. In both LLC and hepa1‐6 bearing mice, we found that the DNase I decreased the concentrations of IL‐6, IL‐10, IL‐21, and IL‐17, indicating that NETs dismantling might reduce inflammation in mice (Fig. S2). In addition, NETs removal decreased tumor growth significantly (Fig. 4C,D). The results showed that liraglutide and Isotype control, or combination therapy and αPD‐1 presented approximative anti‐tumor effects in NETs‐removal mice (Fig. 4E,F), indicating that NETs play vital roles in the alleviation of tumor progression by liraglutide treatment.

### Combining liraglutide and PD‐1 blockade improved the anti‐tumor activity of CD8 ^+^ T cells

**Fig. 5:**
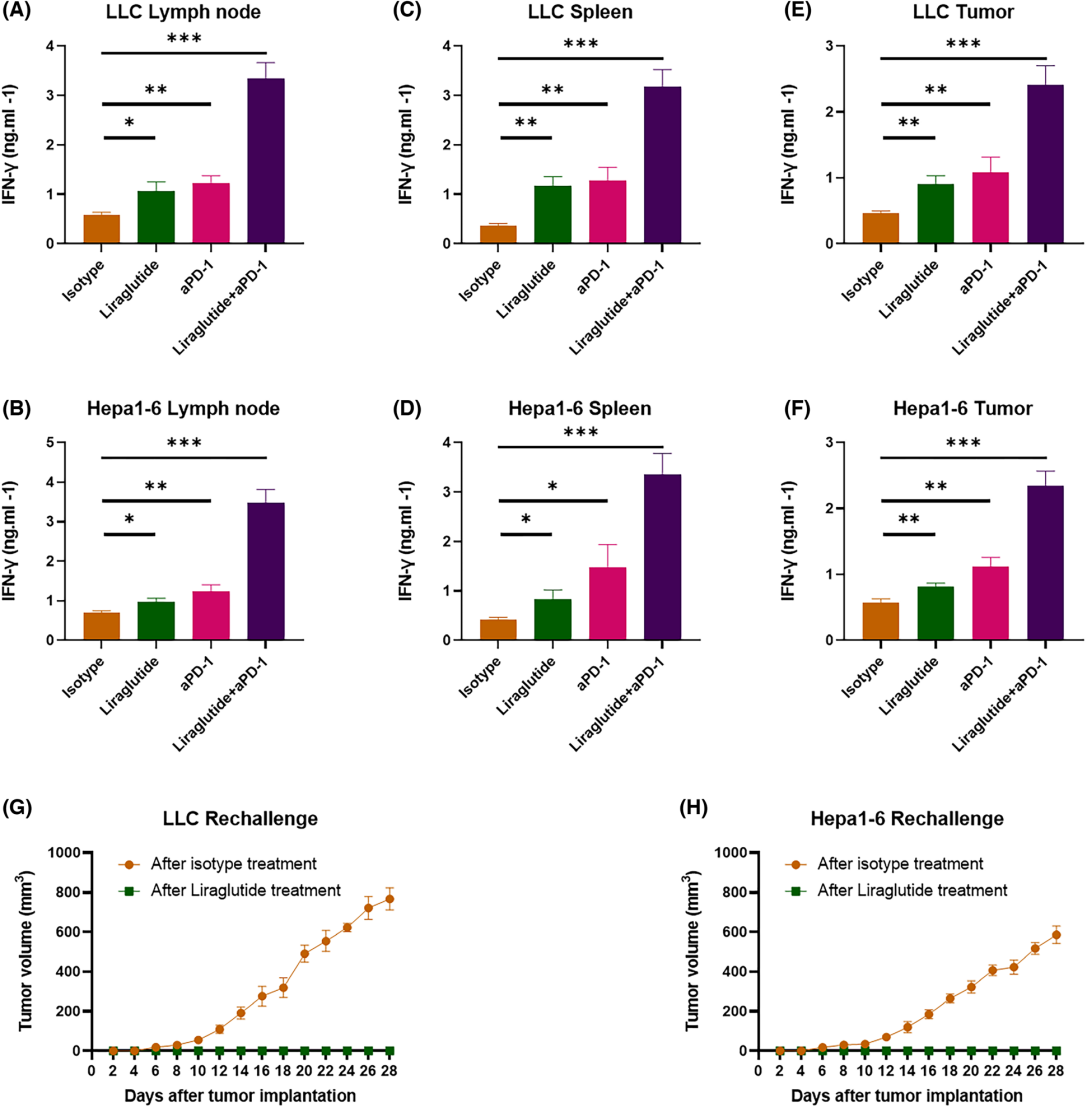
Long‐term immune responses induced by the combination therapy rejected rechallenge. The measurement of IFN‐γ concentration in the culture media of CD8^+^ T cells from (A, B) lymph node, (C, D) spleen, and (E, F) tumor tissues in LLC or Hepa1‐6 allograft mice after 7 days of treatment. *N* = 12 mice for every group. (G) LLC or (H) Hepa1‐6 allograft C57BL/6 mice were treated with STR. Mice pre‐treated with PBS, or the combination therapy received tumor rechallenge by administration of respective cell lines. *N* = 6 mice for every group. Tumor volume = 0.5 × length × width^2^. Statistical significance was determined with two‐tailed *t*‐test. Nonparametric tests were utilized to assess statistical significance between different treatment groups. *P < 0.05, **P < 0.01, ***P < 0.001. Error bars represent SD.

Because CD8^+^ T cells play essential roles against malignancy in immunotherapy, we further explored the impact of Liraglutide+αPD‐1 combination therapy on CD8^+^ T cells, enriching tumor‐infiltrated CD8^+^ T cells for further research. Due to the importance of IFN‐γ in CD8^+^ T cell‐mediated cytotoxicity, we measured the concentration of IFN‐γ secreted by the enriched CD8^+^ T cells post‐stimulation with irradiated LLC or Hepa1‐6. We found that the combination therapy increased the cytotoxicity of CD8^+^ T cells from lymph nodes (Fig. 5A,B), spleen (Fig. 5C,D), and tumor bulks (Fig. 5E,F) significantly. To determine whether the Liraglutide+αPD‐1 combination therapy induced long‐term antitumor CD8^+^ T cell responses, we conducted surgical resection of primary tumors on one side of the mice and rechallenged them through administration of respective tumor cell lines on the opposite side. We found that the tumor formation disappears completely in combination therapy‐treated mice compared to the mice pre‐treated with PBS (Fig. 5G,H). These findings indicate that the Liraglutide+αPD‐1 combination therapy induced long‐term anti‐tumor immunity capable of protecting the tumor rechallenged mice.

## Discussion

Liraglutide is widely used in treating type 2 diabetes through binding to the GLP‐1 receptor, decreasing pancreatic beta‐cell apoptosis, and promoting cell proliferation [2]. However, liraglutide also influences the biological behavior of other cell types, such as reducing neutrophil extravasation and activation [2, 7, 9]. To explore the impact of liraglutide on peripheral blood cells, we measured cells using complete blood counting. Interestingly, we found that in lung and liver cancer models, liraglutide decreased neutrophil number.

Considering the impact of liraglutide on neutrophil activation and the critical roles of neutrophils in cancer prognosis [51], we further analyzed the important circulating molecules released by neutrophils, finding that liraglutide decreased NETs markers myeloperoxidase, elastase, and dsDNA in both tumor models. Furthermore, because neutrophils mainly influenced tumor progression in the tumor microenvironment, we further explored whether liraglutide modulates tumor‐infiltrated NETs. Interestingly, the NETs formation in tumor tissues also decreased after liraglutide treatment.

Neutrophils have multiple effects on tumor progression, and NETs attract increasing attention in cancer research [52]. The elevated levels of NETs in pancreatic cancer are associated with shorter survival [53, 54, 55]. Some studies have tried to measure the circulating levels of NETs markers in the serum of cancer patients [56]. In this study, we first found that liraglutide inhibits NETs formation in circulation and tumor microenvironment. To some extent, this study indicates the importance of reducing NETs in delaying tumor progression by liraglutide.

Notably, ROS is involved in NETs formation, and serum ROS concentrations are associated with circulating NETs [57]. Liraglutide reduced NETs formation as well as ROS production in tumor infiltrated neutrophils. Therefore, we further performed *in vitro* study to explore the role of ROS in liraglutide‐reduced NETs. PMA was a stimulant often employed to induce NETs through the induction of ROS [42, 58, 59, 60]. Therefore, it was used in our study for the initiation of NETs. In our research, NETs formation in PMA‐treated neutrophils was decreased by liraglutide. However, the ROS inducer Diallyl tetrasulfide reversed the inhibition of NETs formation by liraglutide, further confirming the hypothesis that liraglutide decreased NETs formation by reducing ROS level. Our results were consistent with previous studies that liraglutide could downregulate the generation of ROS [39, 40, 41], and ROS were important mediators in NETs formation [21, 38]. In addition, we further proved that liraglutide inhibits NETs formation by decreasing ROS production. Of course, in future we will use a non‐cancer line for baseline, especially for studying the influence of liraglutide in ROS. This is more convincing for exploring the effect of liraglutide on ROS.

During NETs formation, DNA becomes decorated with granule proteins, such as elastase and MPO [18, 61]. Neutrophil‐stimulation results in rapid NADPH oxidase activation and increased intracellular ROS [62]. Increased ROS mobilizes the cytoskeleton to transport particles outside the activated neutrophils [63]. Our results showed that liraglutide inhibited ROD‐dependent NETs formation. The possible mechanism is liraglutide restrained NADPH oxidase activation, leading to decreased ROS production and NETs formation.

It has been reported that the presence of NETs was associated with a worse prognosis in Ewing sarcoma [64]. Also, NETs inhibition improves the antitumoral effect of PD‐1 blockade in pancreatic cancer [26]. We further explored whether liraglutide influenced the efficacy of anti‐PD‐1 in treating cancers, finding that the combination of liraglutide and anti‐PD‐1 surpassed single treatment in restricting tumor growth and prolonging survival. Of course, this is not enough to validate whether NETs are involved in the inhibition of tumor progression by liraglutide because the decrease of NETs might be the bystander effect. Therefore, we removed NETs in liraglutide‐treated tumor mice using DNase I. As a result, NETs depletion reduced tumor suppression effects by liraglutide. Based on these explorations, we draw the preliminary conclusion that NETs play direct roles in attenuating tumor progression by liraglutide.

It is documented that CD8^+^ T cells play vital roles in suppressing tumor development [65, 66, 67] and are the primary effectors in PD‐1 blockade‐induced antitumor responses [68, 69, 70, 71]. In our study, the combination therapy of liraglutide and PD‐1 targeting increased IFN‐γ secretion from CD8^+^ T cells in the tumor, lymph nodes, and spleen. The evidence indicated that liraglutide presented systemic effects in tumor mice, consistent with the results that both peripheral and tumor infiltrated neutrophils were influenced by liraglutide. Furthermore, the impact of liraglutide was not only systemic but also long‐lasting. After surgical resection, the liraglutide‐treated mice rejected tumor rechallenge effectively. In this study, NETs might form a physical and functional barrier that shields tumor cells from CD8^+^ T cells, favoring tumor growth due to a lack of immune recognition. On the contrary, liraglutide might expose tumor cells to CD8^+^ T cells by eliminating NETs, thereby causing CD8^+^ T cell killing and memory of tumor cells.

In summary, we identified liraglutide as a NETs‐reduced regent in lung and liver cancers and elucidated that the suppression of ROS is an essential mediator. Notably, the liraglutide‐reduced NETs improved the efficacy of PD‐1 blockade in treating cancers. Here, we explore critical insights for tumor development and provide clues for enhancing the effects of immune checkpoint blockade.

## Conflict of interest

The authors declare no conflict of interest.

## Author contributions

DC designed and performed the experiments, analyzed the data, wrote the paper, and replied to comments. HL and LH performed the experiments and revised the paper. ZW and HZ designed the research, revised the paper, and approved the final manuscript.

## Supporting information


**Fig. S1.** Confirmation of depletion of NETs in LLC or Hepa1‐6 bearing mice. Circulating dsDNA was purified and the concentration was measured using ultramicrospectrophotometer. Nonparametric tests were utilized to assess statistical significance between different treatment groups. **P < 0.01. ***P < 0.001. Error bars represent SD. N = 3 mice for every group. Triple assays were performed.
**Fig. S2.** Measurement of inflammation associated cytokines concentrations after depletion of NETs. Plasma was collected from LLC or Hepa1‐6 bearing mice after dismantling NETs using DNase I. The concentrations of (A) IL‐6, (B) IL‐21, (C) IL‐10 and (D) IL‐17 were measured using ELISA. Nonparametric tests were utilized to assess statistical significance between different treatment groups. *P < 0.05. **P < 0.01. ***P < 0.001. Error bars represent SD. N = 5 mice for every group. Triple assays were performed.

## Data Availability

The data that support the findings of this study are available.
