# Supplementary material for: Liraglutide enhances the effect of checkpoint blockade in lung and liver cancers through the inhibition of neutrophil extracellular traps
Source: FEBS Open Bio. 2024 Jun 28;14(8):1365–77. doi: 10.1002/2211-5463.13499 (PMC11301266; doi:10.1002/2211-5463.13499)
Supplement: Supplementary file 1 — Fig. S1. Confirmation of depletion of NETs in LLC or Hepa1‐6 bearing mice. Circulating dsDNA was purified and the concentration was measured using ultramicrospectrophotometer. Nonparametric tests were utilized to assess statistical significance between different treatment groups. **P < 0.01. ***P < 0.001. Error bars represent SD. N = 3 mice for every group. Triple assays were performed. Fig. S2. Measurement of inflammation associated cytokines concentrations after depletion of NETs. Plasma was collected from LLC or Hepa1‐6 bearing mice after dismantling NETs using DNase I. The concentrations of (A) IL‐6, (B) IL‐21, (C) IL‐10 and (D) IL‐17 were measured using ELISA. Nonparametric tests were utilized to assess statistical significance between different treatment groups. *P < 0.05. **P < 0.01. ***P < 0.001. Error bars represent SD. N = 5 mice for every group. Triple assays were performed. [file FEB4-14-1365-s001.docx]

**Supplementary Figure 1.** Confirmation of depletion of NETs in LLC or Hepa1-6 bearing mice. Circulating dsDNA was purified and the concentration was measured using ultramicrospectrophotometer. Nonparametric tests were utilized to access statistical significance between different treatment groups. ***P* < 0.01. ****P* < 0.001. Error bars represent SD. N = 3 mice for every group. Triple assays were performed.


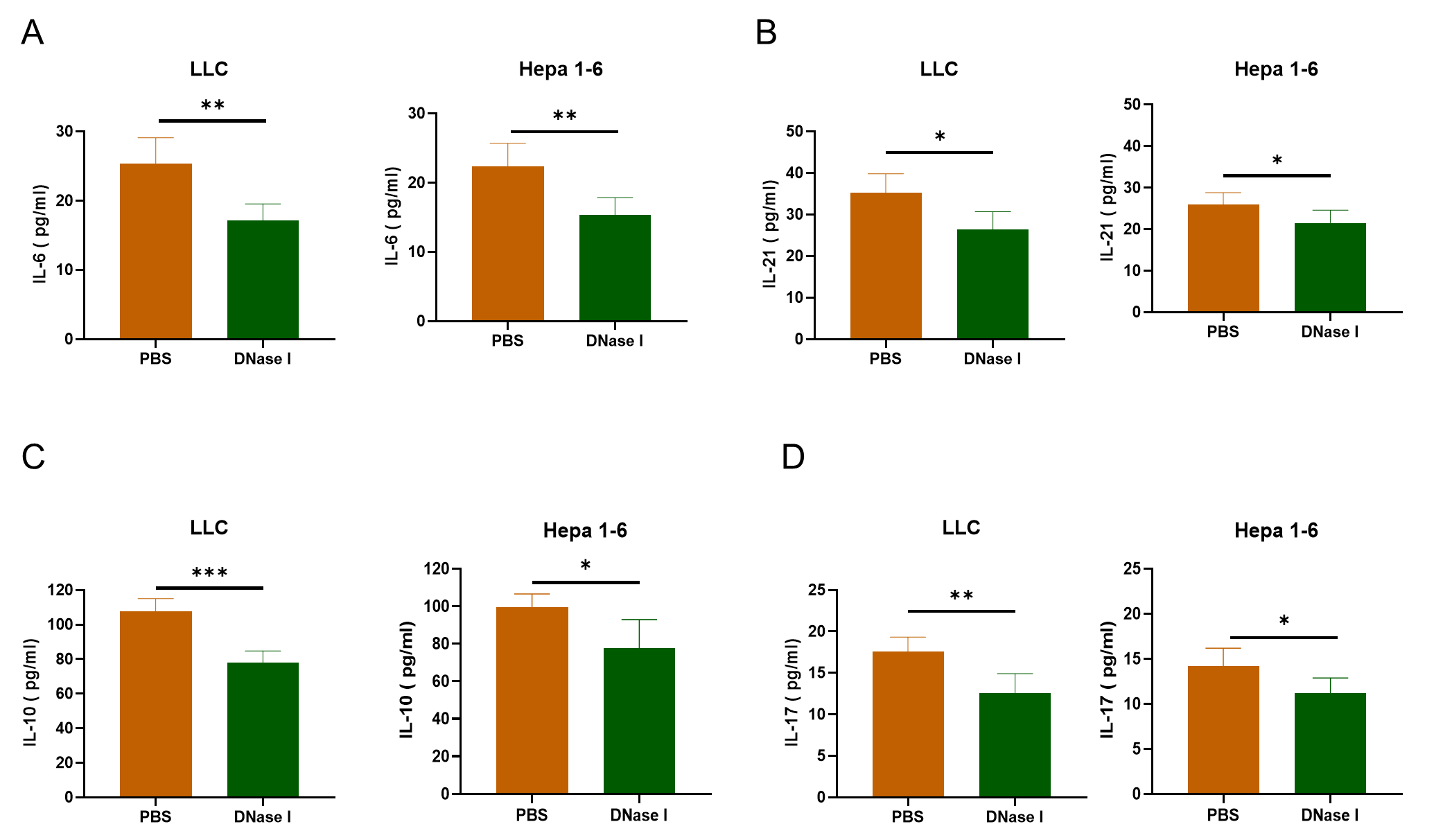


**Supplementary Figure 2.** Measurement of inflammation associated cytokines concentrations after depletion of NETs. Plasma was collected from LLC or Hepa1-6 bearing mice after dismantling NETs using DNase I. The concentrations of (A) IL-6, (B) IL-21, (C) IL-10 and (D) IL-17 were measured using ELISA. Nonparametric tests were utilized to access statistical significance between different treatment groups. *P < 0.05. **P < 0.01. ***P < 0.001. Error bars represent SD. N = 5 mice for every group. Triple assays were performed.
